# Efficacy of Corticosteroids and Intravenous Immunoglobulins in a Patient with Toxic Epidermal Necrolysis Secondary to Sulfadoxine: A Case Report and Literature Review

**DOI:** 10.3390/reports6030035

**Published:** 2023-07-31

**Authors:** Alba Escolà-Rodríguez, Ángel Marcos-Fendian, Carla Bastida, Javier Gil Lianes, Pedro Castro, José Manuel Mascaró, Dolors Soy Muner

**Affiliations:** 1 Pharmacy Service, Division of Medicines, Hospital Clínic of Barcelona, University of Barcelona, 08036 Barcelona, Spain; 2 Department of Dermatology, Hospital Clínic of Barcelona, University of Barcelona, 08036 Barcelona, Spain; 3 Medical Intensive Care Unit, Hospital Clínic of Barcelona, IDIBAPS, University of Barcelona, 08036 Barcelona, Spain

**Keywords:** toxic epidermal necrolysis, sulfadoxine, critical care, dermatologic emergencies, adverse reactions, drug-induced toxicoderma

## Abstract

Toxic epidermal necrolysis (TEN) is a rare life-threatening mucocutaneous reaction characterized by epidermal detachment. Treatment success relies on early diagnosis, rapid withdrawal of the causative drug and supportive care. However, clinical evidence for therapeutic management and specific treatment is insufficient and controversial. We describe the successful management of a TEN case secondary to sulfadoxine managed in our intensive care unit. The patient presented a generalized exanthema with mucocutaneous detachment affecting 45% of the body surface area, positive Nikolsky sign, perianal enanthema and conjunctival hyperemia. Treatment with intravenous immunoglobulins and corticosteroids was prescribed, as well as calcium folinate to prevent myelotoxicity of the causative drug. In this case, hemodialysis was dismissed due to the low efficiency of this technique in removing the triggering drug. Our case report confirms the efficacy of corticosteroids, IGIV, topical treatment on mucocutaneous lesions and supportive care for the management of TEN secondary to sulfadoxine.

## 1. Introduction

Toxic epidermal necrolysis (TEN), or Lyell’s syndrome, is an adverse immunological mucocutaneous reaction characterized by extensive epidermal necrosis and skin detachment. It is defined as a rare disease, with an estimated incidence of 0.4–1.4 cases/million people/year. With a mortality rate from 25 to 70% and a high associated morbidity [1], it is considered the most severe cutaneous adverse reaction (SCAR) [2].

Drugs are the most common trigger of TEN, with allopurinol, antibiotics (especially sulfonamides and penicillins), antiepileptic drugs (carbamazepine, phenobarbital and phenytoin) and non-steroidal anti-inflammatory drugs (NSAIDs) being the most frequently involved agents [1,2,3,4,5]. Infections, including *Mycoplasma pneumoniae* infection, are the second most common trigger of TEN, particularly in children [6]; in over one-third of TEN cases, the causal factor cannot be identified [7].

The severity and mortality rates of TEN can be evaluated by applying prognostic scoring systems, such as the severity-of-illness score for TEN (SCORTEN). It is based on seven clinical and laboratory variables assessed in the first 24 h of admission. The SCORTEN score ranges from 0 to 7, with higher scores indicating a greater risk of mortality. Notably, a score of above 5 corresponds to a mortality rate over 90% [1,2,4].

Treatment success depends on early diagnosis, rapid withdrawal of the causative drug and the instauration of supportive care. Several systemic immunosuppressive or immunomodulating treatments (SITs), such as corticosteroids, intravenous immunoglobulin (IGIV), cyclosporine and etanercept, have been proposed, as well as extracorporeal treatments (dialysis and plasmapheresis). However, the evidence for these treatments is limited and controversial [8,9], with the combined therapy of corticosteroids and IGIV demonstrating a significant reduction in mortality in a recent meta-analysis [10].

The main aim of this report is to highlight this rare severe mucocutaneous reaction secondary to sulfonamide antibiotics and to describe the therapeutic management that was successfully carried out.

## 2. Detailed Case Description

### 2.1. Medical History

A 55-year-old African-American male, with unknown drug allergies or toxic habits and no past medical history of interest, was admitted to the emergency department.

The patient frequently travels to Africa for business, and he had recently been to Benin for one month. On this trip, he was prescribed oral antimalarial prophylaxis with atovaquone/proguanil (250 mg/100 mg), which he started two days before travelling. On the fourth day of treatment (day +2 of the trip), he discontinued antimalarial prophylaxis due to gastrointestinal adverse effects. Two days later (day +4), he initiated a new prophylactic treatment with sulfadoxine/pyrimethamine (500 mg/25 mg), for which he took three tablets every 24 h for 21 days instead of one tablet weekly, leading to an overdosage of 21-fold.

On day +25, a routine blood test detected residual malaria. Thus, an oral antimalarial treatment with sulfamethoxypyrazine/artesunate/pyrimethamine (500 mg/200 mg/25 mg, one tablet every 24 h for 3 days) was started. After 24 h, he presented pruritus and skin erythema, and, consequently, his antimalarial treatment was replaced with artemether/lumefantrine (20 mg/120 mg, four tablets at 0, 8, 24, 36, 48 and 60 h). The following day (day +27), the patient presented clinical worsening with the presence of a macular erythematous rash on the trunk and conjunctivitis; therefore, antimalarial treatment was discontinued.

**Figure 1:**
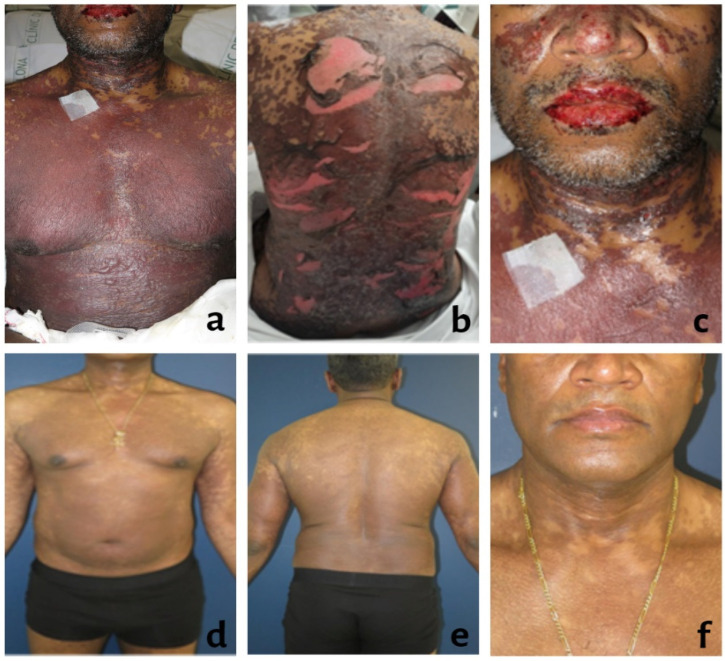
(**a**–**c**) Toxic epidermal necrolysis secondary to sulfadoxine. Confluent erythematous macular exanthema affecting 45% of the body surface area, with areas of skin detachment and the presence of flaccid bullae. At the oral area, there are superficial erosions with crusting. (**d**–**f**) Evolution after 9 months. Resolution of inflammatory lesions without signs of epidermal necrosis, with significant residual post-inflammatory hyperpigmentation.

On day +28, the patient travelled back home and, two days later, due to significant worsening of the skin lesions, he consulted the emergency department. On arrival, he was conscious, oriented and febrile (38 °C) with high blood pressure (160/100 mmHg). Physical examination revealed the presence of confluent and generalized erythematous purpuric macules of various sizes and shapes that began to coalesce, affecting 45% of the body surface area (BSA), epidermal detachment with the formation of flaccid bullae at the pectoral region and positive Nikolsky sign. Mucosae were also affected with labial crusted erosions, perianal enanthema, conjunctival hyperemia and well-defined ulcers over the glans penis (Figure 1a–c).

With the suspicion of a TEN, the patient was admitted to our intensive care unit (ICU) for continuous monitoring and supportive care. On admission, the severity-of-illness score for TEN (SCORTEN) was 2, which is related to a 12.1% mortality rate [3,4]. Laboratory tests showed an increase in acute-phase reactants (C-reactive protein (CRP) 7.72 mg/dL and procalcitonin 0.8 ng/mL), serum creatinine 1.33 mg/dL, lactate dehydrogenase (LDH) 448 UI/L, ferritin 1310 ng/mL, D-dimer 2120 ng/mL, moderate liver cytolysis with aspartate aminotransferase (AST) 105 UI/L and alanine aminotransferase (ALT) 124 UI/L and normal coagulation parameters. Microbiological cultures, blood microfilariae, Stongyloides serology and thick blood smear for malaria were negative. The chest X-ray was normal. The skin biopsy disclosed a subepidermal bullae along with epidermal necrosis and perivascular lymphoid infiltrate. Moreover, direct immunofluorescence tested negative, supporting the diagnosis of TEN.

#### 2.1.1. Investigations

Whereas infections are the second most common trigger of TEN, especially *Mycoplasma pneumoniae* and human herpesvirus infections, *Plasmodium* spp. infections have not yet been reported as a direct trigger of TEN. However, drugs used to treat or to prevent this infection, such as sulfadoxine/pyrimethamine, have been described as one of the most common causes of TEN [4,6].

The most important factor to improve patient outcomes is the identification and withdrawal of the culprit drug. To identify it, we considered the most common drugs associated with TEN and the temporary relationship with the onset of the disease that usually occurs 7–21 days after starting the triggering medication [4]. We reviewed the treatments the patient received and found a probable causal correlation with sulfadoxine/pyrimethamine, mainly due to two circumstances: the timing of symptom presentation (3 weeks after starting the drug) and a higher association of TEN with antibiotics of the sulfonamide group. In addition, the adverse drug reaction probability scale described by Naranjo et al. revealed a probable causal relationship of sulfadoxine/pyrimethamine, with a score of 7 [11]. Sulfamethoxypyrazine was dismissed as the causative agent because the patient had only taken one dose the day before the appearance of the first symptoms. Moreover, a lymphocyte proliferation test (LPT) was performed the day after admission, being positive for sulfadoxine and negative for pyrimethamine and artesunate. Thus, a definite diagnosis of TEN secondary to sulfadoxine was made.

In the clinical interview, it was detected that the patient had mistakenly taken an overdosage of sulfadoxine/pyrimethamine 21-fold higher than the recommended dose for malaria prophylaxis [9]. Although TEN is an idiosyncratic reaction, considered as not dose-dependent, the literature reports a higher prevalence of this disorder in patients who received multiple doses of the drug compared with patients who received a single drug dose [12], suggesting that drug overexposure could have an impact on the onset of TEN. The potential adverse effects of overexposure to this drug were reviewed.

#### 2.1.2. Treatment

The patient was treated with IGIV (0.5 g/kg/day for 4 days), methylprednisolone (1 mg/kg/day for 7 days) and empirical intravenous antibiotic therapy with ceftriaxone (1 g/12 h) and clindamycin (600 mg/8 h) for 7 days. No adverse effects related to these treatments were noticed.

We decided to use the combined regimen of corticosteroids plus IGIV as it showed the most significant evidence for a reduction in mortality according to a Tsai et al. meta-analysis [10]. Since there are no comparative studies showing a higher efficacy of pulsed corticosteroid therapy, we chose to treat the patient with methylprednisolone at a dose of 1 mg/kg/day.

Mucocutaneous lesions were treated with daily topical dressings: cleansing with 0.9% sodium chloride followed by the magistral-formulation subacetate of lead (Goulard’s water) on genitals and potassium permanganate 1/10.000 on denuded areas. Sodium fusidate cream was applied to the genital and lip lesions, and sterile liquid Vaseline was applied to non-denuded but necrotic areas. Topical treatment with silver sulfadiazine cream was discarded, as it contains sulfamide. Finally, all erosive areas were covered with dressings of balsam of Peru and castor oil. Perioral enanthema was treated with an in situ magistral formulation of mucositis solution (nystatin suspension 100.000 UI/mL (60 mL), 2% lidocaine (10 mL), gentamicin 80 mg (2 mL), hydrocortisone 100 mg (1 mL), bicarbonate 1/6 M (to 500 mL)) and liquid diet. In addition, strict pain management was carried out with intravenous paracetamol, diazepam, morphine and fentanyl according to the patient’s tolerance. Conjunctival hyperemia was treated with anti-inflammatory eye drops (1% prednisone (1 drop/4 h)) and antibiotic eye drops (0.3% ofloxacin (1 drop/8 h)).

Since sulfadoxine and pyrimethamine are folic acid antagonists, both drugs can cause myelotoxicity, especially pyrimethamine. To avoid this potential adverse effect, the administration of intravenous calcium folinate was initiated at a dose of 15 mg/day for 8 days [13]. No signs of myelotoxicity were detected under this treatment.

In our case, extracorporeal techniques were not used. Hemodialysis was dismissed due to the low efficiency of this technique in removing the triggering drug, and plasmapheresis was considered only in the case of unfavorable evolution or no response to corticosteroids and IGIV treatment, which never happened.

### 2.2. Outcome and Follow-Up

On day 6 of ICU admission, the patient evolved favorably with no progression of cutaneous necrosis and the appearance of re-epithelialization areas. Twelve days after admission, the patient was transferred to the dermatology ward with complete cutaneous re-epithelialization, improvement in oral and genital mucosal lesions and photophobia and ocular chemosis reduction. After 14 days, he was finally discharged from the hospital.

At present, 9 months after the clinical manifestation, the patient remains asymptomatic, without xerosis or lesions on the oral mucosa, and with only several residual coalescent hyperpigmented macules on the trunk and extremities (Figure 1d–f). The ocular involvement has not yet resolved, and the patient remains under treatment for conjunctival hyperemia.

## 3. Discussion

TEN is a severe mucocutaneous reaction that requires careful clinical and pharmacotherapeutic care. The management of TEN is based on early identification and withdrawal of the causative drug, as well as supportive care to preserve hemodynamic stability and prevent complications [5,8,9]. Although the literature provides evidence on case series and observational studies with different specific treatments for this disorder, including SITs and extracorporeal treatments, the clinical evidence is still insufficient and controversial [1,3,5], with the combined therapy of corticosteroids and IGIV being an alternative that demonstrated a significant reduction in mortality in a recent meta-analysis [10].

Sulfadoxine/pyrimethamine contains a sulfonamide antibiotic, reported to be one of the most frequently associated drugs with TEN [3,4,14]. Patients that received multiple doses of the drug, which is generally used in the prevention of malaria, are at higher risk of TEN than those who received a single dose, which is generally used for the treatment of malaria [12]. In the present case, sulfadoxine/pyrimethamine was identified as the causative agent through a temporal correlation since the onset of symptoms occurred 21 days after starting this treatment and for containing one of the drugs typically associated with this entity [4]. Overexposure to this drug may have played a role in the onset of TEN. Furthermore, according to the Naranjo et al. adverse reaction probability scale criteria, the causal relationship of this drug-induced adverse effect was considered as probable [11]. Finally, the use of LPT was essential to confirm the culprit drug. LPT can identify the triggering agent in 50–80% of cases, and it is an important tool in the investigation of the causative drug in multi-treated patients [15].

TEN is a life-threatening disorder with a high mortality rate (25–70%) [1]. Garcia-Doval et al. showed a better prognosis, with an increased rate of survival, for patients with early withdrawal of the culprit drug, especially when it has a short elimination half-life [16]. Sulfadoxine and pyrimethamine have a long elimination half-life, about 100 h for pyrimethamine and 200 h for sulfadoxine [13], which could translate into a higher risk of mortality.

As the patient took a 21-fold overdose of sulfadoxine/pyrimethamine, extracorporeal techniques were evaluated. El-Azhary et al. described three cases of patients with SCAR, in which hemodialysis therapy was crucial in reversing the adverse drug reaction [17]. Hydrosolubility, low plasma protein binding and small molecular weight are physicochemical and pharmacokinetic characteristics that determine drug removal via this technique. In our case, this technique was dismissed since both sulfadoxine and pyrimethamine are highly protein bound (approximately 90%) [13]. Narita et al. reported that plasmapheresis was effective in patients with TEN who were refractory to supportive care or systemic corticosteroid therapy [18]. In our patient, plasmapheresis was also evaluated, but, considering the initial favorable evolution with IGIV, corticosteroid and supportive care, it was finally discarded.

New promising alternative treatments with cyclosporine and etanercept have been proposed [9,10,19,20,21]. Patel et al. performed a meta-analysis comparing the results of 24 studies (*n* = 979), where they found that cyclosporine could reduce mortality in TEN patients [19]. On the other hand, Jacobsen et al. observed, in a systematic review, that etanercept may result in a reduction in mortality compared with systemic corticosteroids [20]. In our case, cyclosporine therapy was discarded because of its potential hematologic toxicity [22], and etanercept was not used due to limited efficacy evidence.

## 4. Conclusions

Our case report describes the efficacy of corticosteroids, IGIV, topical treatment on mucocutaneous lesions and supportive care for the management of TEN. Further research and clinical evidence are needed to develop optimal treatment guidelines for the appropriate care of these patients.

## Data Availability

Not applicable.
